# Potential novel therapeutic strategies in cystic fibrosis: antimicrobial and anti-biofilm activity of natural and designed α-helical peptides against *Staphylococcus aureus*, *Pseudomonas aeruginosa*, and *Stenotrophomonas maltophilia*

**DOI:** 10.1186/1471-2180-12-145

**Published:** 2012-07-23

**Authors:** Arianna Pompilio, Valentina Crocetta, Marco Scocchi, Stefano Pomponio, Valentina Di Vincenzo, Mario Mardirossian, Giovanni Gherardi, Ersilia Fiscarelli, Giordano Dicuonzo, Renato Gennaro, Giovanni Di Bonaventura

**Affiliations:** 1 Department of Biomedical Sciences, “G. d’Annunzio” University of Chieti, Via Vestini 31, 66100 Chieti, Italy; 2 Center of Excellence on Aging, “G. d'Annunzio” University Foundation, Via Colle dell’Ara, 66100 Chieti, Italy; 3 Department of Life Sciences, University of Trieste, Via L. Giorgieri 1, 34127 Trieste, Italy; 4 Center for Integrated Research, “Campus Biomedico” University, Via A. Del Portillo, 00128 Rome, Italy; 5 "Bambino Gesù" Children's Hospital and Research Institute, Piazza Sant’Onofrio 4, 00165 Rome, Italy

**Keywords:** Cystic fibrosis, Antimicrobial peptides, Biofilm

## Abstract

**Background:**

Treatment of cystic fibrosis-associated lung infections is hampered by the presence of multi-drug resistant pathogens, many of which are also strong biofilm producers. Antimicrobial peptides, essential components of innate immunity in humans and animals, exhibit relevant in vitro antimicrobial activity although they tend not to select for resistant strains.

**Results:**

Three α-helical antimicrobial peptides, BMAP-27 and BMAP-28 of bovine origin, and the artificial P19(9/B) peptide were tested, comparatively to Tobramycin, for their *in vitro* antibacterial and anti-biofilm activity against 15 *Staphylococcus aureus*, 25 *Pseudomonas aeruginosa*, and 27 *Stenotrophomonas maltophilia* strains from cystic fibrosis patients. All assays were carried out in physical-chemical experimental conditions simulating a cystic fibrosis lung. All peptides showed a potent and rapid bactericidal activity against most *P. aeruginosa, S. maltophilia* and *S. aureus* strains tested, at levels generally higher than those exhibited by Tobramycin and significantly reduced biofilm formation of all the bacterial species tested, although less effectively than Tobramycin did. On the contrary, the viability-reducing activity of antimicrobial peptides against preformed *P. aeruginosa* biofilms was comparable to and, in some cases, higher than that showed by Tobramycin.

**Conclusions:**

The activity shown by α-helical peptides against planktonic and biofilm cells makes them promising “lead compounds” for future development of novel drugs for therapeutic treatment of cystic fibrosis lung disease.

## Background

Physicians treating patients with cystic fibrosis (CF) are increasingly faced with infections caused by multidrug-resistant strains. *Pseudomonas aeruginosa* and *Staphylococcus aureus* are the most common bacterial pathogens isolated from the CF respiratory tract where they cause persistent infections associated with a more rapid decline in lung function and survival [1,2]. In recent years, however, there has been an increasing number of reports on potentially emerging and challenging pathogens, probably due to improved laboratory detection strategies and to selective pressure exerted on bacterial populations by the antipseudomonal antibiotic therapy [2]. In this respect, both the overall prevalence and incidence of intrinsically antibiotic-resistant *Stenotrophomonas maltophilia* isolations from CF respiratory tract secretions have been recently reported [3-5].

Efforts to treat CF infections are also hampered by the high microbial adaptation to the CF pulmonary environment, resulting in an increased ability to form biofilms intrinsically resistant to therapeutically important antibiotics such as aminoglycosides, fluoroquinolones, and tetracycline [6-10].

Novel antimicrobial agents that could replace or complement current therapies are consequently needed to fight chronic infections in CF patients.

Antimicrobial peptides (AMPs) are naturally occurring molecules of the innate immune system that play an important role in the host defence of animals and plants [11-13]. Over the last years, natural AMPs have attracted considerable interest for the development of novel antibiotics for several reasons [14,15]: i) the broad activity spectrum, comprised multiply antibiotic-resistant bacteria; ii) the relative selectivity towards their targets (microbial membranes); iii) the rapid mechanism of action; and, above all, iv) the low frequency in selecting resistant strains. Although the antimicrobial activity of AMPs has been extensively reported in literature [13-17], only few studies have been reported with respect to CF pathogens [18-21].

Hence, in an attempt to evaluate the therapeutic potential of AMPs in the management of CF lung infections, for the first time in the present study three cationic α-helical AMPs - two cathelicidins of bovine origin (BMAP-27, BMAP-28) and the artificial peptide P19(9/B) - were tested for their in vitro antibacterial effectiveness, as well as their *in vitro* anti-biofilm activity, against selected *S. aureus*, *P. aeruginosa*, and *S. maltophilia* strains collected from CF patients. The efficacy of the AMPs was compared to that of Tobramycin, selected as the antibiotic of choice used for chronic suppressive therapy in CF patients.

Since the conditions present in the CF patients’ airway surface liquid could counteract the potency of antibiotics such as Tobramycin [22,23], in the present study all *in vitro* antimicrobial assays were carried out under experimental conditions simulating the physical-chemical properties observed in CF lung environment [24-26].

## Results

### Phenotypic features and clonal relatedness of CF strains

A total of 9 out of 25 *P. aeruginosa* strains tested showed mucoid phenotype on MHA, while 3 exhibited SCV phenotype. Among 15 *S. aureus* isolates tested, 7 were methicillin-resistant.

PFGE analysis showed 8, 21, and 12 different pulsotypes among *S. aureus*, *S. maltophilia*, and *P. aeruginosa* isolates, respectively. Among *S. aureus* isolates, only the PFGE type 1 was shared by multiple strains, which comprised 8 isolates and 7 PFGE subtypes. Among *S. maltophilia* isolates, 2 multiple-strains PFGE types were observed: PFGE type 23 (5 isolates, 2 PFGE subtypes), and PFGE type 73 (2 isolates with identical PFGE profile). Among *P. aeruginosa* isolates, 5 multiple-strains PFGE types were observed: PFGE type 5 (6 isolates, 2 PFGE subtypes), PFGE type 1 (4 isolates with indistinguishable PFGE profile), PFGE types 9 and 11 (3 isolates each, with identical PFGE pattern), and PFGE type 8 (2 isolates, one PFGE subtype) (data not shown).

### *In vitro *activity of AMPs and Tobramycin against planktonic cells: MIC, MBC

In order to determine the efficacy of AMPs, the antimicrobial activity was measured against 67 CF clinical isolates, and results are summarized in Table 1. Overall, BMAP-28 showed the widest activity spectrum among AMPs tested, as suggested by MIC_90_ and MBC_90_ values (16 μg/ml, for both), although all of them exhibited a species-specific activity. In fact, although AMPs showed comparable activity against *P. aeruginosa*, BMAP-28 was found to be more active than P19(9/B) against *S. maltophilia*, and resulted the best active AMP against *S. aureus* (MIC_90_: 32 μg/ml; MBC_90_: 32 μg/ml). Compared to AMPs, Tobramycin exhibited a lower activity (MIC_90_ and MBC_90_: >64 μg/ml) regardless of the species considered. Killing quotient values, calculated as MBC/MIC ratio, were < 4 for all AMPs**,** as well as for Tobramycin, clearly suggesting a bactericidal activity. No differences in susceptibility levels to AMPs were found with regard to phenotype (mucoid, SCV, MRSA), pulsotype, or susceptibility to Tobramycin (data not shown).

**Table 1:** *In vitro*** activity of BMAP-27, BMAP-28, P19(9/B), and Tobramycin against*P. aeruginosa, S. maltophilia*and*S. aureus*CF strains**

**Bacterial strains (n)**	**Test agent:**			
	**BMAP-27**	**BMAP-28**	**P19(9/B)**	**TOBRAMYCIN**
***P. aeruginosa*(25)**				
MIC_50_^a^	8	16	8	16
MIC_90_^b^	16	32	32	>64
MIC_range_	4-16	4–32	4–32	2- > 64
MBC_50_^c^	8	16	16	32
MBC_90_^d^	16	32	64	>64
MBC_range_	4–16	4–64	4- > 64	2- > 64
MBC/MIC	1.3	1.2	1.9^e^	1.5^f^
***S. maltophilia* (27)**				
MIC_50_^a^	4	4	4	>64
MIC_90_^b^	8	4	16	>64
MIC_range_	4-8	2–8	4–32	4- > 64
MBC_50_^c^	8	4	8	>64
MBC_90_^d^	16	8	32	>64
MBC_range_	4–32	2–16	4–64	8- > 64
MBC/MIC	1.9	1.3	1.7	1.3^g^
***S. aureus*(15)**				
MIC_50_^a^	64	8	64	>64
MIC_90_^b^	>64	32	>64	>64
MIC_range_	32- > 64	4–32	32- > 64	4- > 64
MBC_50_^c^	>64	8	>64	>64
MBC_90_^d^	>64	32	>64	>64
MBC_range_	64- > 64	4–32	32- > 64	4- > 64
MBC/MIC	1.2^h^	1.2	1.2^i^	1.0^l^
**Total (67)**				
MIC_50_^a^	8	4	8	>64
MIC_90_^b^	>64	16	64	>64
MIC_range_	4->64	2–32	4- > 64	2- > 64
MBC_50_^c^	8	8	16	>64
MBC_90_^d^	>64	16	>64	>64
MBC_range_	4- > 64	2–64	4- > 64	2- > 64
MBC/MIC	1.5^m^	1.2	1.7^n^	1.4^o^

^a, b^MIC_50_ and MIC_90_: MIC (μg/ml) inhibiting 50 and 90% of the strains tested, respectively.

^c, d^ MBC_50_ and MBC_90_: MBC (μg/ml) eradicating 50 and 90% of the strains tested, respectively.

Only isolates exhibiting in range MIC values were considered for killing quotient calculation (MBC/MIC): ^e^n = 24; ^f^n = 12; ^g^n = 3; ^h^n = 6; ^i^n = 2; ^m^n = 58; ^n^n = 57;^o^n = 17.

MIC and MBC values obtained under CLSI-recommended or “CF-like” experimental conditions (see Materials and Methods section) are shown in Table 2. Comparative evaluation of these values showed that mean MIC_CF-like_/MIC_CLSI_ and MBC_CF-like_/MBC_CLSI_ values obtained for Tobramycin (23.9 and 15.6, respectively) were significantly higher than those observed for BMAP-27 (1.5 and 1.2, respectively; *p* < 0.001), BMAP-28 (0.5 and 0.5, respectively; *p* < 0.001), and P19(9/B) (2.8 and 2.9, respectively; *p* < 0.001), regardless of species tested, indicating a reduced antibiotic activity of Tobramycin in CF-like conditions.

**Table 2:** **Antimicrobial activity of BMAP-27, BMAP-28, P19(9/B) and Tobramycin evaluated under different experimental conditions: “CF-like” (5% CO**_**2**_**, pH 6.8, SCFM) and “standard CLSI-recommended” (aerobiosis, pH 7.2, CAMHB)**

**Bacterial strains**	**Susceptibility (MIC**_**CF-like**_**/MIC**_**CLSI**_**) to:**			
	**BMAP-27**	**BMAP-28**	**P19(9/B)**	**TOBRAMYCIN**
***P. aeruginosa***				
Pa1	8/4	8/8	4/16	4/0.25
Pa5	8/4	16/16	8/8	16/2
Pa6	8/8	16/16	16/8	8/8
Pa9	8/4	16/16	16/8	64/1
Sm109	4/8	4/16	4/8	128/64
Sm126	8/16	8/32	4/32	256/64
Sm143	8/8	4/8	4/4	8/2
***S. aureus***				
Sa1	128/64	8/16	128/16	256/64
Sa3	64/64	4/32	64/16	256/16
Sa4	64/64	4/16	32/8	32/2
Sa7	64/16	4/16	64/8	256/2
**Mean MIC**_**CF-like**_**/MIC**_**CLSI**_	1.5	0.5	2.8	23.9
***P. aeruginosa***				
Pa1	8/8	8/16	16/32	4/1
Pa5	16/8	16/32	16/16	16/4
Pa6	16/8	16/16	16/32	8/8
Pa9	8/8	16/32	64/16	128/2
Sm109	8/16	8/16	8/8	256/128
Sm126	8/32	16/32	8/32	256/64
Sm143	16/8	8/8	4/4	8/8
Sa1	128/64	8/16	128/16	256/64
Sa3	64/64	4/32	64/16	256/32
Sa4	64/64	8/32	32/8	32/2
Sa7	64/ND^a^	8/16	64/8	256/4
**Mean MBC**_**CF-like**_**/MBC**_**CLSI**_	1.2	0.5	2.9	15.6

^a^ ND, not determined.

### Bactericidal kinetics

**Figure 1:**
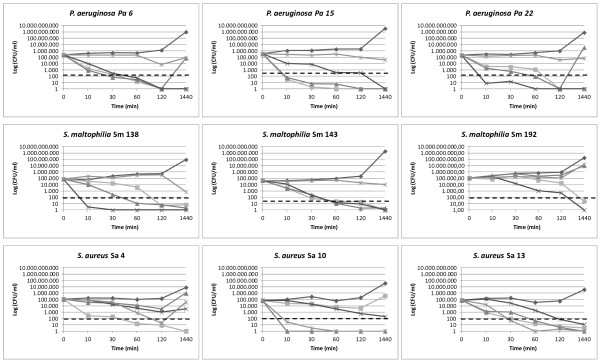
**Time-killing kinetic of AMPs against CF strains.** BMAP-27 (■), BMAP-28 (▴), P19(9/B) (×), and Tobramycin () were tested at MIC value against representative *P. aeruginosa* (Pa6, Pa15, and Pa22), *S. maltophilia* (Sm138, Sm143, and Sm192), and *S. aureus* (Sa4, Sa10, and Sa13) CF strains. Controls (♦) were not exposed to drugs. Values are the mean of two independent experiments performed in triplicate. The dotted line indicates a 3-log reduction in viability.

Time-killing results have been summarized in Figure 1. BMAP-27, BMAP-28, and P19(9/B) exerted a rapid bactericidal activity against *P. aeruginosa*, reducing the number of viable bacterial cells of at least 3 logs within 60 min of exposure. However, the bactericidal effect of BMAP-28 against *P. aeruginosa* was incomplete for two (Pa6 and Pa22) of the three strains tested, allowing bacterial regrowth after 24-h incubation, although at levels lower than those observed for untreated control. In parallel experiments, Tobramycin showed only a bacteriostatic effect against *P. aeruginosa,* causing no more than 1-log reduction in viable count after 24 h.

BMAP-27, BMAP-28 and P19(9/B) exerted bactericidal activity also against *S. maltophilia*, although with streaking strain-specific differences. Particularly, BMAP-28 exhibited only bacteriostatic effect against Sm192 strain, while P19(9/B) showed a rapid bactericidal effect against Sm138 strain, causing more than a 4-log reduction in viable count after 10 min-exposure. Tobramycin exhibited a late (after 24-h exposure) bactericidal effect only against Sm138 strain.

AMPs activity against *S. aureus* was significantly strain-specific, ranging from the rapid bactericidal activity of BMAP-28 against Sa10 strain, to the bacteriostatic effect of P19(9/B) and BMAP-28 against Sa4 strain. Tobramycin showed a bactericidal effect against all *S. aureus* strains tested, although allowing bacterial regrowth of Sa4 strain after 2-h exposure.

### *In vitro* activity of Tobramycin-AMP combinations against planktonic cells

Results from checkerboard assays are summarized in Table 3. FICI values showed that all AMP + Tobramycin combinations tested showed an indifferent effect against *P. aeruginosa* and *S. maltophilia* strains. Conversely, BMAP-27 + Tobramycin (tested at 16 + 8, 16 + 4, and 16 + 2 μg/ml, respectively) combination exhibited synergic effect against Sa4 strain (the only one tested, 100% synergy), while P19(9/B) + Tobramycin (tested at 4 + 2, 4 + 1, and 8 + 1 μg/ml, respectively) combination exhibited synergic effect against *S. aureus* Sa10 strain (1 out of 3 strains tested, 33.3% synergy).

**Table 3:** **In vitro effect of AMP + Tobramycin (TOB) combinations against*P. aeruginosa*,*S. maltophilia*, and*S. aureus*CF strains**

**Drug combinations**	***P. aeruginosa***			***S. maltophilia***			***S. aureus***		
	**Synergy**	**Indifference**	**Antagonism**	**Synergy**	**Indifference**	**Antagonism**	**Synergy**	**Indifference**	**Antagonism**
	**FICI**^**a**^**≤ 0.5**	**0.5 < FICI ≤ 4**	**FICI > 4**	**FICI ≤ 0.5**	**0.5 < FICI ≤ 4**	**FICI > 4**	**FICI ≤ 0.5**	**0.5 < FICI ≤ 4**	**FICI > 4**
**BMAP-27 + TOB**	0 (0%)	12 (100%)	0 (0%)	0 (0%)	8 (100%)	0 (0%)	1 (100%)^b^	0 (0%)^b^	0 (0%)^b^
**BMAP-28 + TOB**	0 (0%)	12 (100%)	0 (0%)	0 (0%)	8 (100%)	0 (0%)	0 (0%)^c^	1 (100%)^c^	0 (0%)^c^
**P19(9/B) + TOB**	0 (0%)	12 (100%)	0 (0%)	0 (0%)	8 (100%)	0 (0%)	1 (33.3%)^d^	2 (66.7%)^d^	0 (0%)^d^

^a^ Fractional Inhibitory Concentration Index (FICI).

Only isolates exhibiting in-range MIC values were considered for checkerboard titration method: *P. aeruginosa* (n = 12), *S. maltophilia* (n = 8), and *S. aureus* (^b^ n = 1; ^c^ n = 1; ^d^ n = 3).

### In vitro activity of AMPs and Tobramycin against biofilm

All CF strains were screened for biofilm forming ability on polystyrene. A significantly higher proportion of biofilm producer strains was found in *P. aeruginosa* and *S. aureus*, compared to *S. maltophilia* (96 and 80% vs 55%, respectively; *p* < 0.01) (data not shown). However, efficiency in biofilm formation was significantly higher in *P. aeruginosa* than in *S. aureus*, as suggested by median biofilm amounts produced (0.162 vs 0.109, respectively; *p* < 0.01) (data not shown).

**Figure 2:**
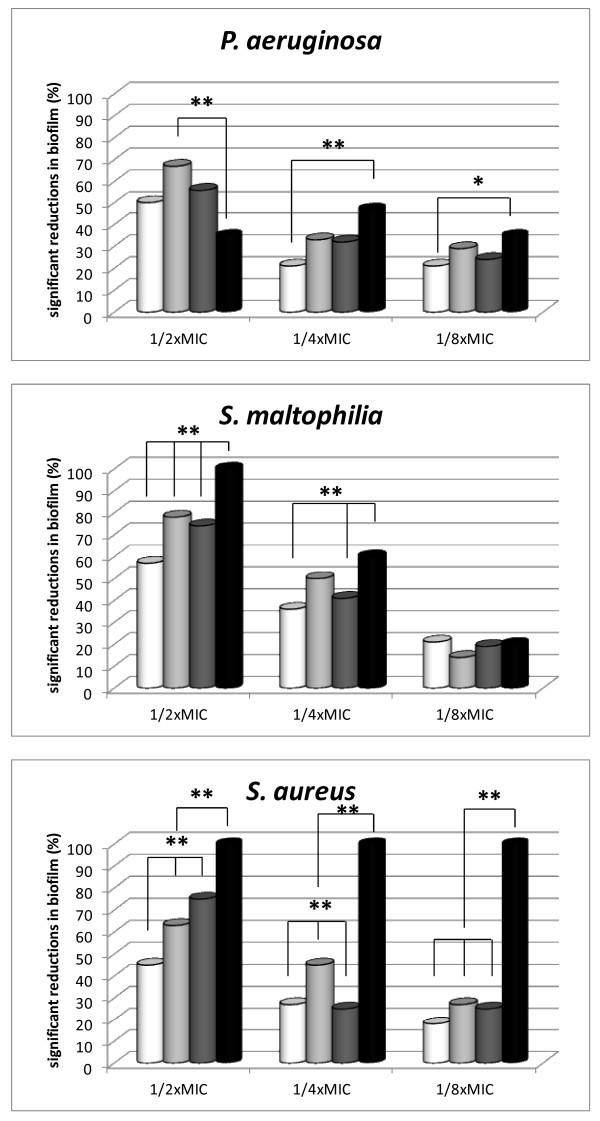
**Effect of AMPs at sub-inhibitory concentrations against biofilm formation by CF strains.** BMAP-27 (white bars), BMAP-28 (light gray bars), P19(9/B) (dark gray bars), and Tobramycin (black bars) were tested at 1/2x, 1/4x, and 1/8xMIC against biofilm formation by *P. aeruginosa* (n = 24, 24, 25, and 17, for BMAP-27, BMAP-28, P19(9/B) and Tobramycin, respectively)*, S. maltophilia* (n = 14, 14, 27, and 5, for BMAP-27, BMAP-28, P19(9/B) and Tobramycin, respectively)*,* and *S. aureus* (n = 11, 11, 8, and 3, for BMAP-27, BMAP-28, P19(9/B) and Tobramycin, respectively) CF strains. Prevention of biofilm formation was plotted as percentage of strains whose ability in forming biofilm was significantly decreased (of at least 25%) compared to controls (not exposed), as analyzed by a crystal violet staining assay.* *p* < 0.05; ** *p* < 0.0001, Fisher’s exact test.

To determine if AMPs could be prophylactically used to prevent biofilm formation, we tested the effect of AMPs and Tobramycin at sub-inhibitory concentrations (1/2x, 1/4x, and 1/8xMIC) against biofilm formation (Figure 2). Tobramycin at 1/2x and 1/4xMIC caused a significantly higher reduction in biofilm-forming ability of *S. maltophilia* and *S. aureus*, in comparison with the three AMPs. This effect was more relevant with *S. aureus*, being observed also at 1/8xMIC. Tobramycin showed to be more effective than BMAP-27 against *P. aeruginosa* at concentrations equal to 1/4x and 1/8xMIC. The activity of Tobramycin in reducing biofilm formation was not related to drug susceptibility (data not shown). Among AMPs, BMAP-28 and P19(9/B) at 1/2xMIC were significantly more active compared to BMAP-27, and BMAP-28 at 1/4xMIC was significantly more active than other AMPs against *S. aureus*.

**Figure 3:**
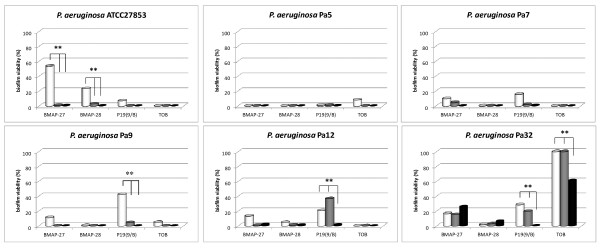
**Activity of AMPs at bactericidal concentrations against preformed*P. aeruginosa*biofilms.** BMAP-27, BMAP-28, P19(9/B), and Tobramycin were tested at 1x (white bars), 5x (gray bars), and 10xMIC (black bars) against preformed biofilm by 6 *P. aeruginosa* CF strains. Results are expressed as percentage of biofilm’ viability compared to control (not exposed, 100% viability). ** *p* < 0.0001, Fisher’s exact test.

We further evaluated AMPs as potential therapeutics for CF by testing their efficacy against preformed biofilms. To this, BMAP-27, BMAP-28, P19(9/B), and Tobramycin at 1xMIC and at bactericidal concentrations (5x, and 10xMIC) were assayed against preformed (24 h) biofilms by six representative *P. aeruginosa* strains selected for high biofilm formation ability (Figure 3).

The activity of AMPs and Tobramycin against preformed biofilms resulted to be similar in 5 out of 6 strains tested, causing a highly significant reduction of biofilm viability compared to the controls (biofilm not exposed; *p* < 0.0001), regardless of the concentrations tested (Figure 3). AMPs showed to be active at all concentrations, also against biofilms formed by *P. aeruginosa* Pa32, against which Tobramycin was effective only at the highest concentration used (10xMIC). The activity of Tobramycin against preformed biofilms was not related to drug susceptibility (data not shown).

## Discussion

This study was aimed at verifying the potential of some α-helical AMPs as lead compounds for the development of novel antimicrobials to treat lung disease in CF patients. To this, we tested the *in vitro* susceptibility of *P. aeruginosa*, *S. maltophilia* and *S. aureus* CF isolates to the naturally occurring AMPs BMAP-27 and BMAP-28, as well as the rationally designed P19(B/9), and we compared their effectiveness with that of Tobramycin, the antibiotic of choice for the inhalation therapy of chronic airway infections in CF patients.

BMAP-27 and BMAP-28 are two cathelicidin-derived peptides of bovine origin that have a role in innate defence [27,28]. The hallmark of cathelicidins is the presence of a conserved N-terminal proregion associated with C-terminal antimicrobial sequences showing a remarkable diversity and considerable inter-species differences [13]. BMAP-27 and BMAP-28 are cationic (charge: +11 and +8, respectively) and both adopt an α-helical structure on interaction with the negatively charged bacterial surface [28]. Recent results have suggested that AMPs with these characteristics may be the most effective against strains producing exogenous polysaccharides that are known to inhibit the activity of other types of AMPs [19,29]. For this reason, we added to our study also a third peptide from this class which has been rationally designed, making use also of non-proteinogenic aminoacids, to optimize its propensity to assume α-helical conformation [30].

Effort to treat CF are also hampered by the conditions present in patients’ airway surface liquid where the accumulation of large volumes of viscous sputum (mucus) providing bacteria with a nutritionally rich growth environment composed of host- and bacterial-derived factors which deeply change their phenotype and possibly their susceptibility against AMPs [31]. Therefore, to accurately judge the feasibility of these peptides as potential anti-infectives in the context of CF, in this study we investigated the activity of AMPs under some CF-like experimental conditions, including acidic pH, reduced O_2_ tension, and a chemically defined medium mimicking the nutritional composition of CF sputum [24-26].

These conditions allow pathogens to assume a physiology similar to that shown *in vivo* in the CF lung [24] and constitute a more realistic model to assay their sensitivity to AMPs.

Evaluation of MIC and MBC values, as well as time-killing assays against planktonic forms of different CF isolates of *P. aeruginosa, S. maltophilia,* and *S. aureus*, have shown that all three AMPs are highly active *in vitro* against most tested strains, although BMAP-28 showed the widest spectrum of activity. It is noteworthy that all the three peptides exhibited an activity higher than Tobramycin. This observation is even more evident when considering the molar concentration (μM) of each compound rather than that by weight (μg/ml), given that the peptides tested are at least six folds heavier than Tobramycin.

The poor activity showed by Tobramycin is probably due to the experimental conditions used in this study, as suggested by comparative evaluation of MIC values observed in both “CF-like” and CLSI-recommended conditions. On the contrary, the activity of AMPs tested resulted to be slightly enhanced (BMAP-28), unaffected (BMAP-27), or slightly reduced [P19(9/B)] in “CF-like” conditions, compared to CLSI-recommended ones, so they can be considered to be quite robust and medium insensitive.

MBC/MIC ratio clearly indicated that all AMPs exert a bactericidal effect against the CF isolates, in agreement with the known capability of BMAP-27, BMAP-28 and P19(B/9) to kill target cells by rapid permeabilization of their membranes [28]. Results of killing kinetic assays confirmed this mode of action, although bactericidal activity against *S. aureus* and *S. maltophilia* was strain-dependent. Again, the potency of AMPs was overall comparable or higher than that showed by Tobramycin.

Due to the different mechanism of action showed by AMPs and Tobramycin, we investigated the potential synergy between them. Interestingly, Tobramycin exhibited synergy with both BMAP-27 and P19(9/B) against planktonic *S. aureus* Sa4 and Sa10 strains, both resistant to Tobramycin, thus suggesting that at least in these cases both AMPs may overcome resistance to Tobramycin by facilitating the internalization of the aminoglycoside into the bacterial cells. Further studies on a more representative number of *S. aureus* strains will be mandatory to understand the mechanism of this synergy and the feasibility to use these AMPs in association with traditional antibiotic treatments.

Within the CF lung, pathogens cells grow as biofilms, which are inherently recalcitrant to antimicrobial treatment and host response [32]. Even worse, it has recently been reported that some antibiotics may even stimulate biofilm formation at subinhibitory concentrations [7]. Biofilm resistance is mainly due to the slow growth rate and low metabolic activity of bacteria in such community. For these reasons, AMPs whose mechanism of action makes them active also on non-growing bacteria, should be able to efficiently inhibit or prevent biofilm formation.

Our results in fact indicate that the three α-helical peptides were all able to reduce biofilm formation, although generally at a less extent than Tobramycin. In particular, all peptides reduced the capacity of *P. aeruginosa, S. maltophilia* and *S. aureus* to form biofilms when used at sub-inhibitory concentrations, with the strongest effects at about 1/2xMIC values, while Tobramycin was efficacious also at lower concentrations (1/4x, and 1/8x MIC). This effect was particular evident with the isolates of *S. aureus*. Interestingly, no planktonic growth inhibition was observed at concentrations able to reduce biofilm formation, and also AMPs with poor killing capacity against some planktonic cells showed anti-biofilm effects. These observations suggest that BMAP-27, BMAP-28 and P19(9/B) may interfere with biofilm formation by different mechanisms other than direct antimicrobial activity similarly to what observed with the human cathelicidin LL-37 [33], and recently reviewed by Batoni et al. [34].

Most CF patients are infected by *P. aeruginosa* whose persistence is due to the formation of antibiotic resistant biofilms in the lung [35]. Our results showed that BMAP-27, BMAP-28, and P19(9/B) were also as effective as Tobramycin in reducing cell viability of preformed biofilms formed by selected strains of *P. aeruginosa.* At MIC concentrations, and even more at 5xMIC values, the two cathelicidins caused highly significant reduction of biofilm viability of all six strains of *P. aeruginosa* whereas Tobramycin showed comparable results only for five isolates. It has previously been reported that extracellular DNA is an important biofilm component [36], and that in *P. aeruginosa* it is involved in cell-cell attachment and biofilm development [37]. Due to the high affinity of cationic AMPs for DNA [38], it may be presumed that this binding might facilitate the detachment or disruption of otherwise-stable biofilm structures.

## Conclusions

The overall results of this study shed new insights on the antibacterial properties of α-helical peptides, allowing the selection of those with the best properties to cope with lung pathogens associated to CF. BMAP-27, BMAP-28 and also the rationally designed P19(9/B) may thus be considered useful not only as lead compounds for the development of novel antibiotics but also for compounds that may counteract bacterial biofilm formation and eradicate preformed biofilms, reflecting the modern understanding of the role of biofilm formation in chronic CF infections. However, before applying these molecules in the future for early prophylactic and therapeutic treatment of CF lung disease, further *in vitro* studies (against other CF pathogens, such as *Burkholderia cepacia*, and fungi), as well as in vivo studies are needed to evaluate their therapeutic potential.

## Methods

### Bacterial strains

Overall, 67 antibiotic-resistant bacterial strains were tested in the present study: 15 *S. aureus*, 25 *P. aeruginosa*, and 27 *S. maltophilia*. Strains were collected from respiratory specimens obtained from patients admitted to the CF Operative Unit, “Bambino Gesù” Children’s Hospital and Research Institute of Rome. Identification to species level was carried out by both manual (API System; bioMérieux, Marcy-L'Etoile, France) and automated (BD Phoenix; Becton, Dickinson and Company, Buccinasco, Milan, Italy) biochemical test-based systems. Each isolate was collected from a single patient and resistant to at least three of the following groups of antibiotics: β-lactams with or without β-lactamase inhibitor, aminoglycosides, fluoroquinolones, folate-pathway inhibitors (trimethoprim-sulphamethoxazole), tetracyclines, and macrolides. Strains were stored at −80°C in a Microbank system (Biolife Italiana S.r.l., Milan, Italy) and subcultured in Trypticase Soya broth (Oxoid S.p.A., Milan, Italy), then twice on Mueller-Hinton agar (MHA; Oxoid S.p.A) prior to the use in this study.

### Phenotypic and genotypic characterization of CF strains

All strains grown on MHA were checked for mucoid phenotype and the emergence of small-colony variants (SCVs). Further, they were screened for their susceptibility to antibiotics by agar-based disk diffusion assay, according to the CLSI criteria [39], and by the Etest following the manufacturer’s instructions assays (Biolife Italiana S.r.l.; Milan, Italy).

All CF strains tested in this study were genotyped by Pulsed-Field Gel Electrophoresis (PFGE) analysis in order to gain clue on genetic relatedness of strains. DNA was prepared in agarose plugs for chromosomal macrorestriction analysis as previously described [40,41]. For *S. aureus* isolates, agarose plugs were digested with enzyme *SmaI* (40U). DNA from *P. aeruginosa* and *S. maltophilia* isolates was digested using *XbaI* (30U). PFGE profiles were visually interpreted following the interpretative criteria previously described [27,40]: in particular, isolates with indistinguishable PFGE patterns were assigned to the same PFGE subtype; for *S. aureus*, isolates differing by 1 to 4 bands were assigned to different PFGE subtypes within the same PFGE type; for *S. maltophilia* and *P. aeruginosa*, isolates were assigned to the same PFGE type with different PFGE subtypes when they differed by 1 to 3 bands.

### Peptide Synthesis, purification and characterization

P19(9/B) (GZZOOZBOOBOOBZOOZGY; where Z = Norleucine; O = Ornithine; B = 2-Aminoisobutyric acid) was a kind gift of Prof. A. Tossi and was prepared as described previously [30]. BMAP-27 (GRFKRFRKKFKKLFKKLSPVIPLLHL-am) and BMAP-28 (GGLRSLGRKILRAWKKYGPIIVPIIRI-am) were synthesised as C-terminal amides by solid-phase peptide Fmoc strategy on a Microwave-enhanced CEM Liberty Synthesizer on a Pal-PEG Rink Amide resin LL (substitution 0.18-0.22 mmol/g). The peptides were purified by RP-HPLC on a Phenomenex preparative column (Jupiter™, C18, 10 μm, 90 Å, 250 × 21.20 mm) using a 20-50% CH_3_CN in 60-min gradient with an 8 ml/min flow. Their quality and purity were verified by ESI-MS (API 150 EX Applied Biosystems). Concentrations of their stock solutions, were confirmed by spectrophotometric determination of tryptophan (ϵ_280_ = 5500 M^-1^ cm^-1^), by measuring the differential absorbance at 215 nm and 225 nm [42] and by spectrophotometric determination of peptide bonds (ϵ_214_ calculated as described by Kuipers and Gruppen [43]).

### “CF-like” experimental conditions

In order to simulate the physical-chemical properties observed in CF lung environment [24-26], all *in vitro* antimicrobial assays against planktonic (MIC, MBC, time-kill kinetics, synergy testing) and sessile (biofilm formation, preformed biofilms) cells were performed in “CF-like” conditions: i) under reduced oxygen concentration (5% CO_2_); ii) at acidic pH (6.8); and iii) in a chemically defined “synthetic CF sputum medium” (SCFM), that mimics the nutritional composition of CF sputum [24]. SCFM was prepared by using Casamino Acids Vitamin Assay (BD Difco) mixture containing each amino acid at concentration not significantly different from that originally described by Palmer and co-workers [24], except for a reduced amount of glycine and ornithine, which were therefore added from ad hoc prepared stock solutions to reach their required concentration.

### Susceptibility testing

MICs and MBCs were determined by microdilution technique, in accordance with CLSI M100-S20 protocol [39], with some modifications. Briefly, serial two-fold dilutions (64 to 0.12 μg/ml) of each AMP and Tobramycin (Sigma-Aldrich S.r.l.; Milan; Italy) were prepared in SCFM at a volume of 100 μl/well in 96-well microtiter plates (Bibby-Sterilin Italia S.r.l.; Milan, Italy). Each well was then inoculated with 5 μl of a standardized inoculum, corresponding to a final test concentration of about 0.5-1 × 10^5^ CFU/well. After incubation at 37°C for 24 h, the MIC was read as the lowest concentration of the test agent that completely inhibited visible growth. To measure the MBC, 100 μl of broth from clear wells were plated on MHA plates, and incubated at 37°C for 24 h. MBC was defined as the lowest concentration of the test agent killing of at least 99.99% of the original inoculum.

To evaluate the impact of “CF-like” experimental conditions on the antimicrobial activity of AMPs and Tobramycin, a set of PFGE-unrelated isolates representative for different levels of susceptibility to Tobramycin (4 *P. aeruginosa*, 3 *S. maltophilia*, and 4 *S. aureus*) was also tested for MIC and MBC values determined under standard CLSI-recommended conditions (i.e., aerobic atmosphere, cation-adjusted Mueller-Hinton broth, and pH 7.2).

### Time-killing assay

Kinetics of AMPs’ and Tobramycin’ activity was evaluated by using the broth macrodilution method against three representative isolates within each tested species. Briefly, the standardized inoculum (1x10^5^ CFU/mL) was exposed to the test agent at 1xMIC in SCFM, and incubated at 37°C. After 10 min, 30 min and 1, 2, and 24-h of incubation, aliquots of each sample were diluted and plated onto MHA, then the viable counts determined after 24-h of incubation at 37°C. Killing curves were constructed by plotting the log CFU/mL versus time.

### Synergy testing

The activity of each AMP combined to Tobramycin against CF strains was evaluated by checkerboard technique by using 96-well polystyrene microplate (Kartell S.p.A., Noviglio, Milan, Italy). Briefly, concentrations of multiple compounds (range: 64–0.12 μg/ml) were combined in standard MIC format along with 5 × 10^5^ CFU/ml of tested. Inoculated microplates were incubated at 37°C for 24 h under 5% CO_2_. At the end of the incubation, for each combination interaction a Fractional Inhibitory Concentration (FIC) index was calculated as follows: FIC index = Σ (FIC_A_ + FIC_B_), where FIC_A_ is the MIC of drug A in the combination/MIC of drug A alone, and FIC_B_ is the MIC of drug B in the combination/MIC of drug B alone. Synergy was defined as a FIC index of ≤0.5, indifference as a FIC index of >0.5 to ≤ 4, and antagonism as a FIC index of > 4.

### *In vitro* activity against biofilm formation

In each well of a 96-well flat-bottom polystyrene tissue-culture microtiter plate (Iwaki; Bibby-Sterilin Italia S.r.l.), 5 μl of a standardized inoculum (1–5 × 10^7^ CFU/ml) were added to 100 μl of SCFM containing test agent at 1/2x, 1/4x, and 1/8xMIC. After incubation at 37°C for 24 h, non-adherent bacteria were removed by washing twice with 100 μl sterile PBS (pH 7.2; Sigma-Aldrich S.r.l.). Slime and adherent cells were fixed by incubating for 1 h at 60°C, and stained for 5 min at room temperature with 100 μl of 1% crystal violet solution. The wells were then rinsed with distilled water and dried at 37°C for 30 min. Biofilms were destained by treatment with 100 μl of 33% glacial acetic acid for 15 min, and the OD_492_ was then measured. The low cut-off was represented by approximately 3 standard deviations above the mean OD_492_ of control wells (containing medium alone without bacteria). The percentage of inhibition was calculated as follows: (1 – OD_492_ of the test/OD_492_ of non-treated control) x 100.

### *In vitro* activity against preformed *P. aeruginosa* biofilms

*In vitro* activity of AMPs and Tobramycin was evaluated against biofilms formed by 6 *P. aeruginosa* strains, selected because strong biofilm-producers. Biofilms were allowed to form in each well of a 96-well flat-bottom polystyrene tissue-treated microtiter plate (Iwaki), as described above. Biofilms samples were then exposed to 100 μl of drug-containing SCFM (prepared at 1x, 5x, and 10x MIC). After incubation at 37°C for 24 h, non-adherent bacteria were removed by washing twice with 100 μl sterile PBS (pH 7.2), and biofilm samples were scraped with a pipette tip following 5-min exposure to 100 μl trypsin-EDTA 0.25% (Sigma-Aldrich S.r.l.). Cell suspension was then vortexed for 1 min to break up bacterial clumps. Bacterial counts were assessed by plating serial 10-fold dilutions of the biofilm cell suspension on MHA plates.

### Statistical analysis

All experiments were performed at least in triplicate and repeated on two different occasions. Differences between frequencies were assessed by Fisher's exact test. Statistical analysis of results was conducted with GraphPad Prism version 4.00 (GraphPad software Inc.; San Diego, CA, USA), considering as statistically significant a *p* value of < 0.05.

## Abbreviations

CF, Cystic Fibrosis; AMPs, Antimicrobial Peptides; MHA, Mueller-Hinton agar; SCVs, Small-Colony Variants; CLSI, Clinical Laboratory Standards Institute; PFGE, Pulsed-Field Gel Electrophoresis; SCFM, Synthetic CF sputum medium; MIC, Minimum Inhibitory Concentration; MBC, Minimum Bactericidal Concentration; CFU, Colony-Forming Unit; FICI, Fractionary Inhibitory Concentration Index.

## Competing interests

The authors declare that they have no competing interests.

## Authors’ contributions

AP, VC, SP and VDV performed susceptibility assay, time-killing assay, synergy testing, and *in vitro* testing against biofilm formation and preformed biofilms. MS, MM, and RG took care of peptide synthesis, purification and characterization, and of SCFM preparation. GG and GD performed PFGE assay. EF collected clinical strains and also took care of their phenotypic characterization. GDB and MS drafted the manuscript, in collaboration with AP, GG, and RG. GDB also carried out the statistical analysis. All authors read and approved the final version.

## References

[B1] Dasenbrook EC Checkley W Merlo CA Konstan MW Lechtzin N Boyle MP Association between respiratory tract methicillin-resistant Staphylococcus aureus and survival in cystic fibrosis JAMA 2010 303 2386 2392 10.1001/jama.2010.791 20551409

[B2] Emerson J Rosenfeld M McNamara S Ramsey B Gibson RL Pseudomonas aeruginosa and other predictors of mortality and morbidity in young children with cystic fibrosis Pediatr Pulmonol 2002 34 91 100 10.1002/ppul.10127 12112774

[B3] de Vrankrijker AM Wolfs TF van der Ent CK Challenging and emerging pathogens in cystic fibrosis Paediatr Respir Rev 2010 11 246 254 10.1016/j.prrv.2010.07.003 21109184

[B4] Emerson J McNamara S Buccat AM Worrell K Burns JL Changes in cystic fibrosis sputum microbiology in the United States between 1995 and 2008 Pediatr Pulmonol 2010 45 363 370 20232473 10.1002/ppul.21198

[B5] Millar FA Simmonds NJ Hodson ME Trends in pathogens colonising the respiratory tract of adult patients with cystic fibrosis, 1985–2005 J Cyst Fibros 2009 8 386 391 10.1016/j.jcf.2009.08.003 19740710

[B6] Di Bonaventura G Prosseda G Del Chierico F Cannavacciuolo S Cipriani P Petrucca A Superti F Ammendolia MG Concato C Fiscarelli E Casalino M Piccolomini R Nicoletti M Colonna B Molecular characterization of virulence determinants of Stenotrophomonas maltophilia strains isolated from patients affected by cystic fibrosis Int J Immunopathol Pharmacol 2007 20 529 537 17880766 10.1177/039463200702000311

[B7] Hoffman LR D'Argenio DA MacCoss MJ Zhang Z Jones RA Miller SI Aminoglycoside antibiotics induce bacterial biofilm formation Nature 2005 436 1171 1175 10.1038/nature03912 16121184

[B8] Linares JF Gustafsson I Baquero F Martinez JL Antibiotics as intermicrobial signaling agents instead of weapons Proc Natl Acad Sci U S A 2006 103 19484 19489 10.1073/pnas.0608949103 17148599 PMC1682013

[B9] Molina A Del Campo R Maiz L Morosini MI Lamas A Baquero F Canton R High prevalence in cystic fibrosis patients of multiresistant hospital-acquired methicillin-resistant Staphylococcus aureus ST228-SCCmecI capable of biofilm formation J Antimicrob Chemother 2008 62 961 967 10.1093/jac/dkn302 18647744

[B10] Singh PK Schaefer AL Parsek MR Moninger TO Welsh MJ Greenberg E Quorum-sensing signals indicate that cystic fibrosis lungs are infected with bacterial biofilms Nature 2000 407 762 764 10.1038/35037627 11048725

[B11] Lai Y Gallo RL AMPed up immunity: how antimicrobial peptides have multiple roles in immune defense Trends Immunol 2009 30 131 141 10.1016/j.it.2008.12.003 19217824 PMC2765035

[B12] Yang D Biragyn A Kwak LW Oppenheim JJ Mammalian defensins in immunity: more than just microbicidal Trends Immunol 2002 23 291 296 10.1016/S1471-4906(02)02246-9 12072367

[B13] Zanetti M Cathelicidins, multifunctional peptides of the innate immunity J Leukoc Biol 2004 75 39 48 12960280 10.1189/jlb.0403147

[B14] Hancock RE Sahl HG Antimicrobial and host-defense peptides as new anti-infective therapeutic strategies Nat Biotechnol 2006 24 1551 1557 10.1038/nbt1267 17160061

[B15] Zanetti M Gennaro R Skerlavaj B Tomasinsig L Circo R Cathelicidin peptides as candidates for a novel class of antimicrobials Curr Pharm Des 2002 8 779 793 10.2174/1381612023395457 11945171

[B16] Benincasa M Scocchi M Pacor S Tossi A Nobili D Basaglia G Busetti M Gennaro R Fungicidal activity of five cathelicidin peptides against clinically isolated yeasts J Antimicrob Chemother 2006 58 950 959 10.1093/jac/dkl382 17023499

[B17] Brogden KA Antimicrobial peptides: pore formers or metabolic inhibitors in bacteria? Nat Rev Microbiol 2005 3 238 250 10.1038/nrmicro1098 15703760

[B18] Kapoor R Wadman MW Dohm MT Czyzewski AM Spormann AM Barron AE Antimicrobial peptoids are effective against Pseudomonas aeruginosa biofilms Antimicrob Agents Chemother 2011 55 3054 3057 10.1128/AAC.01516-10 21422218 PMC3101385

[B19] Pompilio A Scocchi M Pomponio S Guida F Di Primio A Fiscarelli E Gennaro R Di Bonaventura G Antibacterial and anti-biofilm effects of cathelicidin peptides against pathogens isolated from cystic fibrosis patients Peptides 2011 32 1807 1814 10.1016/j.peptides.2011.08.002 21849157

[B20] Saiman L Tabibi S Starner TD San Gabriel P Winokur PL Jia HP McCray PB Tack BF Cathelicidin peptides inhibit multiply antibiotic-resistant pathogens from patients with cystic fibrosis Antimicrob Agents Chemother 2001 45 2838 2844 10.1128/AAC.45.10.2838-2844.2001 11557478 PMC90740

[B21] Thwaite JE Humphrey S Fox MA Savage VL Laws TR Ulaeto DO Titball RW Atkins HS The cationic peptide magainin II is antimicrobial for Burkholderia cepacia-complex strains J Med Microbiol 2009 58 923 929 10.1099/jmm.0.008128-0 19502364

[B22] Hunt BE Weber A Berger A Ramsey B Smith AL Macromolecular mechanisms of sputum inhibition of tobramycin activity Antimicrob Agents Chemother 1995 39 34 39 10.1128/AAC.39.1.34 7535039 PMC162480

[B23] Mendelman PM Smith AL Levy J Weber A Ramsey B Davis RL Aminoglycoside penetration, inactivation, and efficacy in cystic fibrosis sputum Am Rev Respir Dis 1985 132 761 765 3931522 10.1164/arrd.1985.132.4.761

[B24] Palmer KL Aye LM Whiteley M Nutritional cues control Pseudomonas aeruginosa multicellular behavior in cystic fibrosis sputum J Bacteriol 2007 189 8079 8087 10.1128/JB.01138-07 17873029 PMC2168676

[B25] Song Y Salinas D Nielson DW Verkman AS Hyperacidity of secreted fluid from submucosal glands in early cystic fibrosis Am J Physiol Cell Physiol 2006 290 C741 C749 16207791 10.1152/ajpcell.00379.2005

[B26] Worlitzsch D Tarran R Ulrich M Schwab U Cekici A Meyer KC Birrer P Bellon G Berger J Weiss T Botzenhart K Yankaskas JR Randell S Boucher RC Doring G Effects of reduced mucus oxygen concentration in airway Pseudomonas infections of cystic fibrosis patients J Clin Invest 2002 109 317 325 11827991 10.1172/JCI13870 PMC150856

[B27] Benincasa M Skerlavaj B Gennaro R Pellegrini A Zanetti M In vitro and in vivo antimicrobial activity of two alpha-helical cathelicidin peptides and of their synthetic analogs Peptides 2003 24 1723 1731 10.1016/j.peptides.2003.07.025 15019203 PMC7124310

[B28] Skerlavaj B Gennaro R Bagella L Merluzzi L Risso A Zanetti M Biological characterization of two novel cathelicidin-derived peptides and identification of structural requirements for their antimicrobial and cell lytic activities J Biol Chem 1996 271 28375 28381 10.1074/jbc.271.45.28375 8910461

[B29] Chan C Burrows LL Deber CM Helix induction in antimicrobial peptides by alginate in biofilms J Biol Chem 2004 279 38749 38754 10.1074/jbc.M406044200 15247257

[B30] Pacor S Giangaspero A Bacac M Sava G Tossi A Analysis of the cytotoxicity of synthetic antimicrobial peptides on mouse leucocytes: implications for systemic use J Antimicrob Chemother 2002 50 339 348 10.1093/jac/dkf141 12205058

[B31] Hoiby N Pseudomonas in cystic fibrosis: past, present, and future 1998 Cystic Fibrosis Trust, London, United Kingdom

[B32] Costerton JW Stewart PS Greenberg EP Bacterial biofilms: a common cause of persistent infections Science 1999 284 1318 1322 10.1126/science.284.5418.1318 10334980

[B33] Hell E Giske CG Nelson A Romling U Marchini G Human cathelicidin peptide LL37 inhibits both attachment capability and biofilm formation of Staphylococcus epidermidis Lett Appl Microbiol 2010 50 211 215 10.1111/j.1472-765X.2009.02778.x 20002576

[B34] Batoni G Maisetta G Brancatisano FL Esin S Campa M Use of antimicrobial peptides against microbial biofilms: advantages and limits Curr Med Chem 2011 18 256 279 10.2174/092986711794088399 21110801

[B35] Bjarnsholt T Jensen PO Fiandaca MJ Pedersen J Hansen CR Andersen CB Pressler T Givskov M Hoiby N Pseudomonas aeruginosa biofilms in the respiratory tract of cystic fibrosis patients Pediatr Pulmonol 2009 44 547 558 10.1002/ppul.21011 19418571

[B36] Montanaro L Poggi A Visai L Ravaioli S Campoccia D Speziale P Arciola CR Extracellular DNA in biofilms Int J Artif Organs 2011 34 824 831 10.5301/ijao.5000051 22094562

[B37] Barken KB Pamp SJ Yang L Gjermansen M Bertrand JJ Klausen M Givskov M Whitchurch CB Engel JN Tolker-Nielsen T Roles of type IV pili, flagellum-mediated motility and extracellular DNA in the formation of mature multicellular structures in Pseudomonas aeruginosa biofilms Environ Microbiol 2008 10 2331 2343 10.1111/j.1462-2920.2008.01658.x 18485000

[B38] Hale JD Hancock RE Alternative mechanisms of action of cationic antimicrobial peptides on bacteria Expert Rev Anti Infect Ther 2007 5 951 959 10.1586/14787210.5.6.951 18039080

[B39] Clinical and Laboratory Standards Institute Performance standards for antimicrobial susceptibility texting; sixteenth informational supplement 2010 Clinical and Laboratory Standards Institute,

[B40] Gherardi G De Florio L Lorino G Fico L Dicuonzo G Macrolide resistance genotypes and phenotypes among erythromycin-resistant clinical isolates of Staphylococcus aureus and coagulase-negative staphylococci, Italy FEMS Immunol Med Microbiol 2009 55 62 67 10.1111/j.1574-695X.2008.00499.x 19076222

[B41] Pompilio A Pomponio S Crocetta V Gherardi G Verginelli F Fiscarelli E Dicuonzo G Savini V D'Antonio D Di Bonaventura G Phenotypic and genotypic characterization of Stenotrophomonas maltophilia isolates from patients with cystic fibrosis: genome diversity, biofilm formation, and virulence BMC Microbiol 2011 11 159 10.1186/1471-2180-11-159 21729271 PMC3146419

[B42] Waddell WJ A simple ultraviolet spectrophotometric method for the determination of protein J Lab Clin Med 1956 48 311 314 13346201

[B43] Kuipers BJ Gruppen H Prediction of molar extinction coefficients of proteins and peptides using UV absorption of the constituent amino acids at 214 nm to enable quantitative reverse phase high-performance liquid chromatography-mass spectrometry analysis J Agric Food Chem 2007 55 5445 5451 10.1021/jf070337l 17539659

